# Plasma L-Carnitine and L-Lysine Concentrations in HIV-Infected Patients

**DOI:** 10.2174/1874091X01711010119

**Published:** 2017-12-28

**Authors:** Evgeny V. Butorov

**Affiliations:** The Municipal Center of HIV/AIDS prophylaxis, Surgut, Russian Federation

**Keywords:** HIV, L-carnitine, L-lysine, Viral load, Viral progeny, CD4 count lymphocytes

## Abstract

**Background:**

Virus infections are associated with significant alterations in host cells amino acids profiles that support biosynthetic demands necessary for production of viral progeny. Amino acids play an important role in the pathogenesis of all virus-related infections both as basic substrates for protein synthesis and as regulators in many metabolic pathways.

**Objective:**

Our aim was to determine the changes in plasma L-carnitine levels and its amino acid precursor (L-lysine) in HIV-infected patients.

**Methods:**

We performed a case-control study of 430 HIV-1 infected males (non-vegetarians) without any restriction in the nourishment, before highly active antiretroviral therapy (HAART) and 125 HIV-1 subjects after the introduction of HAART who were periodically monitored in the Municipal Center of HIV/AIDS prophylaxis, Surgut, Russian Federation

**Results:**

The plasma total (TC) and free (FC) L-carnitine concentrations markedly decreased with the clinical stages of HIV infection. The mean plasma TC, FC and L-lysine levels were significantly lower in asymptomatic stage (A) and advanced CDC stages (B, C) HIV-infected patients compared with our reference values. The total and free L-carnitine and its amino acid precursor concentrations mild increased in HIV-infected subjects after the introduction of HAART.

Our data revealed that L-lysine amino acid and its derivative (TC) levels were negatively correlated with viral load and inversely with CD4 count lymphocytes in the total cohort.

**Conclusion:**

The study results show that there was evidence for an association between plasma L-carnitine, L-lysine and HIV-1 RNA levels, immunological markers and clinical stages of HIV infection. The obtained data indicate that level changes of these host essential nutritional elements can play an important role in the HIV life cycle. These findings are important for understanding the pathophysiology of HIV infection and must be considered in further research for the development of new approaches in the treatment of the disease.

## 1 INTRODUCTION

Despite the considerable efforts of the world community and the optimistic expectations of achieving the main goals in response to new cases of HIV-infection (such as universal access to prevention, treatment, care and support) the HIV/AIDS pandemic remains a major health problem. The development of new effective methods and approaches in the treatment of HIV infection in the era of highly active antiretroviral therapy remains an urgent and pressing need. Understanding the pathogenesis of HIV disease and finding points of vulnerability in the relationships between the infecting organism and the virus is the key to achieving full control of HIV infection.

No doubts that viral infections trigger metabolic alterations in host cells that support the energetic and biosynthetic demands of viral replication. It has long been known that infection with viruses leads to general activation of host cell metabolism [1-19], energy dysfunction and significant alterations in protein [20-25] and amino acids profiles [26-30].

Although the same protein catabolic effects in HIV infection have been recognized for a long time [31-37], but there are few data in the literature about infection-related amino acids and their derivatives imbalance in HIV-infected patients [38-46].

Observational studies indicate that amino acids concentrations in the extracellular medium were found to be critical in the life cycle of some viruses and are related to increased risk of diseases rapidly progressing [47-50]. The production of significant amounts of empty virus particles, free of viral nucleic acids has been reported for several virus systems in the absence of essential amino acids *in vitro* [51-55]. In addition, specific plasma aminogram abnormalities have been reported in the context of some infectious processes *in vivo* [26-30, 47].

No doubt, in order for HIV’s replicative cycle to be successful, the virus needs to alter the host cell metabolism to establish an environment that can accommodate the increased demands for nutrients, energy and synthesis of virus particles.

Thus, complete understanding and recognition of the similar effects of HIV interactions with host cell amino acids and their derivatives metabolism are important for understanding of virus biology and getting a new approach for the possible potential effect on the infectious agent.

We assumed that plasma levels of essential L-lysine and its derivative (L-carnitine) have a close relationship with viral and host protein metabolism and they act as indicators to evaluate not only the nutritional status of HIV-infected persons, but also as the prognostic markers of the disease.

The purpose of present study was to investigate possible changes in plasma L-carnitine levels and its precursor in clinical stages HIV-infected patients and to support the hypothesis that essential L-lysine can play an important role in the HIV infection pathogenesis.

## 2 MATERIALS AND METHODS

### 2.1 Patients

We performed a case-control study of 430 HIV-1 infected males (non-vegetarians) without any restriction in the nourishment, before highly active antiretroviral therapy (HAART) and 125 HIV-1 subjects after the introduction of HAART who were periodically monitored in the Municipal Center of HIV/AIDS prophylaxis, Surgut, Russian Federation. We ascertained patients' general attributes (age, sex), medical history, and stage of HIV infection, co-infections and the simultaneous survey period. Blood samples for this study were collected during routine clinical control and analytical monitoring, which included hematological, immunological and virological evaluation.

Clinical stages of the disease were based on the 1993 revised guidelines for HIV infection (Centers for Disease Control and Prevention). All patients without highly active antiretroviral therapy (HAART), were classified into groups I (asymptomatic; A1, A2), II (symptomatic, non-AIDS; B1, B2) and III (AIDS; C1, C2). HIV-infected subjects who were severely immunodepressed (A3, B3 and C3 clinical stages and if CD4 lymphocytes count <200 cells/μl) were excluded from the study.

Patients with the underweight (the body mass index (BMI) less than 18.5) and with clinical manifestations of malabsorption syndrome (diarrhea, weight loss, low levels of serum protein and albumin) were excluded from the study.

**Group 1:** 240 patients were asymptomatic or had slight symptoms, non- and moderately immunodepressed (A1, A2).

**Group 2:** 130 patients were moderately symptomatic and immunodepressed (B1, B2).

**Group 3:** 60 patients were severely symptomatic and moderately immunodepressed (C1, C2).

Comparison group 1:125 subjects after introduction of HAART for more than three years, 30% HIV-patients received nucleoside analog reverse-transcriptase inhibitors (NARTIs) and non-nucleoside reverse-transcriptase inhibitors (NNRTIs) therapy, 5% only on NARTIs and 65% HIV-patients on NNRTIs and protease inhibitors (PIs) therapy (age range: 23-42 years, mean±s.d.: 33±4.2 years).

Comparison group 2:140 healthy regional donors (age range: 21-45 years, mean±s.d.: 34±5.1 years).

Author warrants that the Municipal Center of HIV/AIDS prophylaxis has approved the protocol for any investigation involving humans and that all experimentation was conducted in conformity with ethical and humane principles of research. Blood samples were obtained after informed consent from the patients.

### 2.2 Laboratory Studies

Venous blood samples were drawn between 8 a.m. and 9 a.m. after an overnight fast were collected in 5 ml tubes containing 1.6 mg/ml K^2^ EDTA (BD Vacutainer^®^, USA) and the plasma separated by centrifugation (3500 r.p.m.; 10min). For the analysis of L-lysine and L-carnitine concentrations, plasma samples was deproteinizied with 3% sulphosalicyclic acid, carefully mixed and immediately centrifuged (3500 r.p.m.; 10min) to remove plasma proteins. Aliquots, the supernatant of 100 µl, were pipetted into Eppendorf tubes, stored at -40°C and analyzed within the following fortnight in the same laboratory.

Blood samples for measurement of HIV-1 RNA levels were immediately centrifuged (3500 r.p.m.; 10min), aliquots plasma of 1.0 ml were pipetted into Eppendorf tubes and stored at -40°C till ready for use. For other hematological and immunological parameters, venous blood samples were immediately analyzed at the Center laboratory.

Quantification HIV-1 RNA assay was performed by quantitative competitive (RT-PCR) reverse-transcriptase polymerase chain reaction using the commercially available Amplisens^®^ HIV-monitor-FRT kit (Amplisens^®^, Russian Federation) and real-time PCR cycler (Rotor-Gene Q, QIAGEN, Germany). A laboratory survey of the immune function was conducted by use of a flow cytometer according to the method, defining CD3, CD4 and CD8 lymphocytes count (Navios, Beckman Coulter, USA). The hematological parameters were determined using standard techniques (Biosystems A25, Biosystems S.A., Spain; XP-300, Sysmex Corporation, Japan).

The amino acids were separated by the thin layer chromatography (TLC) method and L-lysine concentrations were detected spectrophotometrically after reaction with ninhydrin reagent. Plasma free and total L-carnitine quantification was measured by an enzymatic UV test kit (Roche Diagnostics GmbH, Germany) adapted to the Biosystems A25 analyzer.

Reference values of plasma L-lysine, free and total L-carnitine were established in apparently healthy donors. Low and high amino acid concentration was considered to be present when plasma L-lysine values were below and above one and a half standard deviations (s.d.) of our mean reference values (80 µmol/l< plasma L-lysine<470 µmol/l). The plasma concentration of free and total L-carnitine in healthy humans was evaluated in the range between 29-50 μmol/L and 32-78 μmol/L, respectively.

No significant differences in plasma amino acid and its derivative concentrations were observed in repeat samples obtained from any given patient. The overall reproducibility of results was consistent to within ±5%.

### 2.3 Statistical Analysis

Plasma L-lysine and L-carnitine values follow a Gaussian distribution (Kolmogorov-Smirnov), so the Student's *t*-test was applied to compare patients' concentrations to reference values, as well as those found in patients in comparison groups, with a 95% confidence interval. The correlation between HIV-1 RNA, immunological markers, amino acid and its derivative concentrations was estimated by a Pearson's correlation coefficient. Data are reported as mean ± s.d. (standard deviation), and *P* values below 0.05 were considered to indicate statistical significance. Statistical analyses were performed using the statistical software package Biostat^®^.

## 3 RESULTS

Clinical data, hematological, immunological and virological status of HIV-infected patients are summarized in Table **1**.

Our data revealed the differences between some hematological parameters in patients in CDC stages of HIV infection. The survey shows a significant decrease in the levels of CD4 count lymphocytes in all clinical stages A (488.7±212.1), B (332.5±238.5) and C (251.2±165.8) patients with HIV compared to controls **(**healthy donors) (1253.7±230.3, *P*<0.001 for all comparison groups; Student's *t*-test). CD4 count lymphocytes markedly decreased with the progression of HIV-infection and we found significant differences of the mean values this immunological parameter between advanced (B, C) and asymptomatic (A) stages HIV-infected subjects (*P*<0.0001 and *P*<0.0001, respectively). The same differences were detected between CD8 count lymphocytes and CD4/CD8 ratio in patients from groups I, II, III and controls (comparison group 1, 2).

We found that HIV-1 RNA plasma levels evidently increased in advanced stages (B, C) of HIV-infection related to the progression of the disease. The mean viral load were significantly higher in clinical stage B (186.700±21.400 s.e.) and C (321.100±44.670 s.e.) subjects compared with values in asymptomatic stage (A) HIV-infected patients (109.300±10.300 s.e., *P*<0.0001 and *P*<0.0001, respectively; Student's t-test).

The levels of TC in asymptomatic stage A (240/117 or 49%), stage B (130/81 or 62%) and stage C (60/41 or 70%) HIV-infected patients were significantly reduced. The same tendencies were found for the plasma concentrations of free L-carnitine: 240/109 or 45% in stage A; 130/77 or 59% and 60/40 or 66% in stages B and C, respectively.

We observed that L-lysine levels decreased with the progression of HIV infection. Low levels of this essential amino acid were detected in 240/139 (58%) of HIV-infected subjects in the asymptomatic stage (A), 130/86 (66%) and 60/40 (67%) in stages B and C, respectively. The lowest levels of L-lysine amino acid were found in stage C HIV-infected patients without antiviral treatment (149.1±110.5).

Our data revealed that the plasma TC and FC concentrations were significantly lower in asymptomatic stage A (33.1±7.8; 23.0±5.2) and advanced stages B (29.4±7.7; 19.2±5.1) and C (26.1±5.8; 16.5±4.9) HIV-infected subjects without HAART compared with healthy subjects (*P*<0.05 or less; Student's *t*-test).

The lowest levels of total, free L-carnitine were found in HIV-infected patients under antiviral treatment (25.7±4.4; 16.2±5.4). No differences were observed between the values of the total and free L-carnitine levels in patients under HAART (comparison group 1) and in subjects before antiretroviral treatment. The present survey shows that the mean TC and FC values in HIV-infected subjects under HAART were significantly lower compared with healthy controls (*P*<0.05 and *P*<0.05, respectively).

In addition, we found that the plasma L-lysine levels markedly decreased with the clinical stages of HIV infection. The mean concentrations of this essential amino acid were significantly lower in advanced stages (B, C) (212.3±135.0 and 149.1±110.5) HIV-infected patients than in healthy subjects (275.8±120.3, *P*<0.005 and *P*<0.001, respectively; Student's *t*-test). At the same time, the plasma concentrations of L-lysine significantly increased in 100/80 (80%) of HIV-infected individuals under HAART. As regards antiretroviral therapy, mean values of this amino acid (283.0±110.7) were significantly higher compared with HIV-infected patients without antiretroviral treatment in stage B and C (*P*<0.05 and *P*<0.001, respectively). No difference was observed between plasma L-lysine levels in the asymptomatic stage (A) HIV-infected subjects, patients receiving antiviral therapy and controls (*P*>0.50).

In addition, we examined that essential amino acid concentrations were positively correlated with its derivatives (TC, FC) (*r*= 0.54, *P*<0.0001; *r*= 0.47, *P*<0.0001; Pearson's correlation coefficient) in the total cohort. The plasma mean values of total, free L-carnitine and L-lysine in HIV-infected subjects are given in Table **2**.

A weak negative correlation was observed between plasma total L-carnitine and viral load in patients without HAART in total cohort (*r*= - 0.14, *P*<0.048) and in advanced clinical stage (B) of HIV disease (*r*= - 0.15, *P*<0.043) and mild negative correlation in stage (C) of HIV infection (*r*= - 0.28, *P*<0.019).

We found the weak and mild significant negative correlation between plasma L-lysine levels and viral load in clinical stage A and B HIV-infected patients (*r*= - 0.19, *P*<0.01 and *r*= - 0.24, *P*<0.02, respectively) and strong negative correlation in stage C (*r*= - 0.43, *P*<0.01). Correlation between plasma L-lysine levels and viral load in entire cohort was *r*= - 0.21, *P*<0.017. The relation between amino acid, total l-carnitine concentrations and HIV-1 RNA levels was not significant in patients under HAART.

The plasma mean values of total L-carnitine, L-lysine and HIV-1 RNA levels in HIV-infected persons are summarized in Table **3**.

The results of our study suggest that there was evidence for an association between plasma L-carnitine, L-lysine and HIV-1 RNA levels, immunological markers and clinical stages of HIV infection.

## 4 DISCUSSION

Plasma amino acids and their derivatives have a close relationship with energy, protein metabolism and body nutrition. Any fluctuation in their normal levels can indicate diseased status or some special physiological condition. They can reflect not only the nutritional status and the metabolic abnormalities, but also act as the prognostic markers of various diseases [56-62].

It is commonly known that considerable changes in the host protein and lipid metabolism, catabolic effects and the negative nitrogen equilibrium lead to alterations in amino acid profiles due to viral infections [3, 5, 10, 14, 26, 33, 34, 47, 63, 64].

Observational studies clearly showed that amino acids deficiency lead to a fundamental change in the metabolism of the infected cells, especially in the control for the production of virus particles [48, 49, 51, 52, 54, 55]. As intracellular parasites with limited energy, construction, and information resources, viruses must rely on the host cell machinery to perform tasks essential for viral replication.

Available data indicate that the concentration of amino acids in intracellular medium can modulate both the dynamics of viral protein synthesis [20, 21, 65] as well as promote the transcription of gene expression [66-71]. The same mechanism is well known in HIV pathogenesis. Human immunodeficiency virus requires a cellular tRNA^Lys^ as a primer for initiation of reverse transcription [72-78]. Meanwhile, the presence of a sufficient concentration of the homonymous covalent L-lysine in HIV-infected cells activates the primer [79-86].

There is strong evidence of the presence, in each viral particle, a number of host tRNA^Lys^ able to make a rapid and guaranteed next replication initiation. HIV virions appear to contain approximately 8 to 25 tRNA^Lys^ molecules per two copies of the capsid’s viral genome [76, 87-89].

Very little data in the literature exist on plasma L-lysine and its derivative changes in HIV disease [38-46]. Available studies have focused on imbalance of L-carnitine (derivative of L-lysine amino acid) in HIV-infected patients under antiviral treatment [43-46]. Several reports indicate that L-carnitine metabolism is greatly disturbed in HIV infection progression and systemic carnitine's deficiency could occur in acquired immunodeficiency disease syndrome (AIDS) [43, 45, 90-92].

This essential factor is involved in many metabolic reactions, such as, fatty acid metabolism and cellular energy production. L-carnitine binds fatty acids, generating various acylcarnitines and implicated in the maintenance of the cellular pool of free coenzyme A (CoA) and in the elimination of potentially toxic acyl-CoA.

Given that L-carnitine plays a key role in cellular homeostasis, its imbalance in HIV-infected subjects would be expected to produce a wide variety of disorders affecting the functions of several organs, including liver, skeletal muscle, heart and brain. L-carnitine metabolism in patients with HIV has not been extensively evaluated.

To date, there are few direct clinical trial data on the effect of changes in the plasma L-carnitine levels and amino acid profiles on immunological and virological markers in HIV-infected subjects [38-46].

From these positions, the study of the amino acid and their derivative profiles is important for understanding the pathophysiology of HIV infection. We hypothesized that constant production of viral proteins requires a huge consumption of essential L-lysine and changes the amino acid and their derivative profiles in patients with HIV.

The findings from previous studies seem to support our hypothesis. In particular, we found that the L-lysine amino acid levels were negatively correlated with viral load [93] and the excess of this amino acid in plasma samples leads to active HIV replication *in vitro* [94].

The purpose of present study was to investigate possible changes in plasma L-carnitine levels and its precursor in clinical stages HIV-infected patients and to support the hypothesis that indispensable L-lysine can play an important role in the HIV infection pathogenesis.

The present study results show that advanced stages of HIV disease are characterized by significant changes of plasma L-carnitine (TC, FC) and L-lysine concentrations.

We observed that plasma total (TC) and free (FC) L-carnitine concentrations markedly decreased with the clinical stages of HIV infection. Reduced levels of L-lysine's derivatives were detected in 240/117 (49%) of HIV-infected subjects (non-vegetarians) in the asymptomatic stage (A), 130/81 (62%) and 60/41 (70%) in the advanced CDC stages (B, C) before HAART.

In addition, we found the same tendencies for the plasma L-lysine's levels. The decrease amino acid concentrations were detected in 58%, 66%, and 67% of HIV-infected subjects in clinical stages A, B and C, respectively, compared with our reference values for similar ages.

The lowest plasma concentrations of essential amino acid were observed among HIV-infected subjects (non-vegetarians) without antiviral treatment in advanced clinical stages of the disease (B, C). The L-lysine concentrations mild increased in patients after the introduction of HAART, compared with patients before treatment in advanced CDC stages (B, C) (*P*<0.05 or less).

At the same time, our study shows that the levels of both total and free L-carnitine markedly decreased with the clinical stages of HIV infection and their lowest levels were found in HIV-infected patients under HAART due to the known facts of drug toxicity [90-92, 95].

The research findings confirm the facts that plasma L-carnitine imbalance has not been identified in people without metabolic disorders, suggesting that humans can synthesize endogenously enough L-carnitine [96]. Even strict vegetarians (or vegans) show no signs of this substrate deficiency, despite the fact that most dietary L-carnitine is derived from animal sources [97].

But at the same time, in our study the mean plasma TC, FC and L-lysine levels were significantly lower in asymptomatic stage (A) and advanced CDC stages (B, C) HIV-infected patients without HAART compared with health controls.

Low concentrations of L-lysine and their derivative, owing to malnutrition or malabsorbtion, is another possible cause, since malabsorbtion and wasting syndrome is a common condition in patients with HIV [95, 98]. However, in present research we have enrolled HIV-infected males (non-vegetarians) without any data, clinical or laboratory symptoms of restriction in the nourishment, which suggested that the protein malnutrition was uncommon in our patients.

Thus, we can assert that these substrates’ changes are not related with dietary limitation derived from special protein-restricted diets, which are the major source of exogenous L-carnitine and absolute source of L-lysine.

Our data revealed that L-lysine amino acid and its derivative (TC) levels were negatively correlated with viral load (*P*<0.05; Pearson's correlation coefficient). We observed the weak and mild significant negative correlation between plasma L-lysine levels and viral load in entire cohort and in clinical stage A and B HIV-infected patients (*P*<0.02 or less) and strong negative correlation in stage C (*P*<0.01).

A weak negative correlation was observed between plasma total L-carnitine and viral load in patients without HAART in total cohort (*P*<0.048) and in advanced clinical stage (B) of HIV disease (*P*<0.043) and mild negative correlation in stage (C) of HIV infection (*P*<0.019). The relation between amino acid, total l-carnitine concentrations and HIV-1 RNA levels was not significant in patients under HAART.

The simultaneous increase of viral load and immune suppression in HIV infection is well known. Thus, the positive correlation between CD4 lymphocytes and levels of plasma L-lysine has been identified in our study.

Our data revealed that L-lysine amino acid and its derivative (TC) levels were negatively correlated with viral load and inversely with CD4 count lymphocytes in the total cohort (*P*<0.05).

The mild significantly positive correlation was observed between plasma L-lysine and CD4 count lymphocytes in patients without antiviral treatment in total cohort patients without HAART and in all clinical stages of HIV disease (A, B and C) (*P*<0.05 or less).

We observed that plasma total L-carnitine levels were correlated with CD4 count lymphocytes in entire cohort HIV-infected subjects without HAART (*P*<0.05; Pearson's correlation coefficient); in patients in advanced stages (B, C) of HIV infection (*P*<0.05 or less) and mild positive correlation in patients under antiviral treatment (*P*<0.05).

No relation was found between the mean values of CD4 lymphocytes and L-lysine in patients under antiviral treatment (*P*>0.05).

There was evidence for an association between plasma L-carnitine, L-lysine and HIV RNA levels, immunological markers and clinical stages of HIV infection. Overall study results indicate that changes of these essential nutritional elements have a close relationship with viral and host cell metabolism and are involved for the production of viral progeny.

In summary, our study seems to support the hypothesis that L-lysine play important role in the synthesis of the virus proteins and in the initiation of HIV's reproduction. It is likely that high concentrations of this essential amino acid in plasma may increase the risk of high HIV RNA levels, subsequent acceleration of immunosuppression and the disease progression. More research is necessary to study the clinical significance of amino acid and their derivative profiles in the complete understanding of viral interactions with host cell metabolism.

## CONCLUSION

The study results show that advanced stages of HIV disease are characterized by significant changes of plasma L-carnitine (TC, FC) and L-lysine concentrations. The levels of these basic substrates are negatively correlated with viral load. We found that the lowest plasma concentrations of essential amino acids and total and free L-carnitine were observed among HIV-infected individuals (non-vegetarians) in advanced clinical stages of the disease (B, C). Determining and tracking the plasma L-lysine and L-carnitine levels could be used for the prognosis and treatment of the patients with HIV infection.

Our results suggest that HIV infection's progression leads to L-carnitine deficiency and we therefore believe that L-carnitine supplementation could have a role as a complementary therapy for HIV-infected patients. Based on the obtained data, we assume that the extra- and intracellular concentrations of L-lysine amino acid play a limiting role in the synthesis of the virus proteins and in transcription initiation of the retrovirus life cycle and the deficiency of this essential nutritional element can reduce the level of viral load.

These findings are important for understanding the pathophysiology of HIV infection and must be considered in further clinical and pilot research for the development of new approaches in the treatment of the disease.

## Figures and Tables

**Table 1:** HIV-infected patients (group I, II, III) and controls (comparison group 1, 2) baseline characteristics.

	**HIV Infection Stage (CDC, 1993)**			**Controls**	
				**Comparison Group 1** **(HIV-infected subjects under HAART)** **(*n*=125)**	**Comparison Group 2** **(healthy donors)** **(*n*=140)**
	**Group I** **(A1, A2)** **(*n*= 240)**	**Group II** **(B2, B2)** **(*n*=130)**	**Group III** **(C1, C2)** **(*n*=60)**		
**Age (years)**	34±5.8	35±5.7	35±5.8	34±4.4	36±5.1
**Male (*n*)**	240	130	60	125	140
**Receiving HAART (*n*)**	0	0	0	125	-
**Duration of HIV (months)**	31±12.3	52±12.2	84±13.7	68±11.5	-
**HIV risk factor (IDU) (*n*)**	240	130	60	125	-
**HIV/HCV** **co-infection (%/*n*)**	93/223	92/120	94/56	93/116	-
**Body mass index (BMI) (kg/m^2^)**	22.75±2.57	22.99±3.32	23.32±2.11	23.84±2.17	22.62±2.43
**Viral charge** ± **(s.e.) (copies/ml)**	109.300± 10.300*	186.700± 21.400*	321.100± 44.670*	0.500±0.45*	-
**CD4 (cells/µl)**	488.7± 212.1^●^	332.5± 238.5*^●^	251.2± 165.8*^●^	424.4± 203.8*″	1253.7± 230.3^●^″
**CD8 (cells/µl)**	1030.2± 477.4^●^	1088.7± 541.8*^●^	1197.7± 383.5*^●^	987.3± 487.4*″	712.4± 303.1^●^″
**CD4/CD8 ratio**	0.45±0.18^●^	0.31±0.21^●^	0.22±0.20*^●^	0.38±0.19*″	1.75±0.14^●^″
**ALT (IU/ml)**	69.5 ± 45.1*^●^	67.2±44.9*^●^	102.3±50.2^●^	131.8±54.0*″	25.0±15.1^●^″
**AST (IU/ml)**	64.0 ± 56.4*^●^	75.6±36.3*^●^	94.5±62.4^●^	102.3±53.7*″	24.0±15.0^●^″
**Bilirubin** (**µmol/l)**	9.7±4.3	10.3±3.9	10.1±4.1	10.2±4.9	7.1±3.2
**Alkaline phosphatase (ALP) (IU/l)**	101.3±83.2^●^	93.5±46.3^●^	98.1±57.6^●^	85.7±44.3″	55.3±36.1^●^″
**Lactate dehydrogenase (LDH) (IU/l)**	338.3±148.1	335.7±125.5	394.1±198.2^●^	352.4±154.8	288.7±81.4^●^
**Creatinine** (**µmol/l)**	89.3±36.1	88.2±28.3	85.2±49.1	89.3±38.0	90.5±33.2
**Gamma-glutamyltrans-ferase (GGT) (IU/l)**	78.2±53.5^●^	92.2±68.5^●^	129.1±84.7^●^	89.4±67.5″	27.7±18.7^●^″
**Urea** (**mmol/l)**	4.77±3.15	4.56±2.81	4.68±3.12	4.69±2.78	4.31±3.12
**Serum protein** **(g/l)**	75.3±5.8	76.2±6.2	75.8±5.3	75.4±6.0	76.8±5.4
**Glucose (mmol/l)**	5.4±0.82	5.5±0.78	5.7±0.75	5.3±0.80	5.3±0.90
**Total cholesterol** **(mmol/l)**	5.40±0.53*	5.72±0.48*	5.76±0.51*	6.82±0.60*″	5.35±0.38″

* Patients without HAART *versus* comparison group 1, *P* < 0.05 or less

^●^ Patients without HAART *versus* comparison group 2, *P* < 0.05 or less

″ Patients under HAART *versus* comparison group 2, *P* < 0.05 or less

Values are given as mean±s.d., except for the viral load (mean±s.e.)

**Table 2:** Plasma L-carnitine and L-lysine concentrations in HIV-infected patients (group I, II, III) and controls (comparison group 1, 2).

	**HIV Infection Stage (CDC, 1993)**			**Controls**	
				**Comparison Group 1** **(HIV-infected subjects under HAART)** **(*n*=125)**	**Comparison Group 2** **(healthy donors)** **(*n*=140)**
	**Group I** **(A1, A2)** **(*n*= 240)**	**Group II** **(B2, B2)** **(*n*=130)**	**Group III** **(C1, C2)** **(*n*=60)**		
**Total carnitine (TC) (μmol/l)**	33.1±7.8^●^	29.4±7.7^●^	26.1±5.8^●^	25.7±4.4″	46.3±5.1^●^″
**Free carnitine (FC) (µmol/l)**	23.0±5.2^●^	19.2±5.1^●^	16.5±4.9^●^	16.2±5.4″	34.1±4.8^●^″
**L-lysine (µmol/l)**	253.1±144.9	212.3±135.0*^●^	149.1±110.5*^●^	283.0±142.7*	275.8±120.3^●^

* Patients without HAART *versus* comparison group 1, *P* < 0.05 or less

^●^ Patients without HAART *versus* comparison group 2, *P* < 0.05 or less

″ Patients under HAART *versus* comparison group 2, *P* < 0.05 or less

Values are given as mean±s.d.

**Table 3:** Comparison plasma total L-carnitine, L-lysine concentrations and HIV-1 RNA levels in HIV-infected patients.

**HIV-Patients**	**HIV-1 RNA (copies/ml) ±s.e.**	**Total L-Carnitine (µmol/l)**	**Pearson's Correlation Coefficient**	**L-lysine (µmol/l)**	**Pearson's Correlation Coefficient**
**The entire cohort *n*=430**	205.700±24.450	27.8±7.1	*r*= - 0.14, *P*<0.048 (S)	224.1±128.3	*r*= - 0.21, *P*<0.017 (S)
**Group I** **(A1, A2)** ***n*=240**	109.300±10.300	33.1±7.8	*r*= - 0.09, *P*>0.05 (NS)	253.1±144.9	*r*= - 0.19, *P*<0.01 (S)
**Group II** **(B1, B2)** ***n*=130**	186.700±21.400	29.4±7.7	*r*= - 0.15, *P*<0.043 (S)	212.3±135.0	*r*= - 0.24, *P*<0.02 (S)
**Group III** **(C1, C2)** ***n*=60**	321.100±44.670	26.1±5.8	*r*= - 0.28, *P*<0.019(S)	149.1±110.5	*r*= - 0.43, *P*<0.01 (S)
**HIV-infected patients under HAART** ***n*=125**	0.500±0.45	25.7±4.4	*r*= - 0.05, *P*>0.05 (NS)	283.0±142.7	*r*= - 0.10, *P*>0.05 (NS)

(S): Differences were considered significant when *P* < 0.05

(NS): not significant

Values are given as mean±s.d., except for the viral load (mean±s.e.)
